# European Medicines Agency Conflicts With the European Food Safety Authority (EFSA) on Bisphenol A Regulation

**DOI:** 10.1210/jendso/bvad107

**Published:** 2023-08-12

**Authors:** R Thomas Zoeller, Linda S Birnbaum, Terrence J Collins, Jerrold Heindel, Patricia A Hunt, Taisen Iguchi, Andreas Kortenkamp, John Peterson Myers, Frederick S vom Saal, Carlos Sonnenschein, Ana M Soto

**Affiliations:** Department of Biology, University of Massachusetts Amherst, Amherst, MA 01003, USA; School of Science and Technology, University of Örebro, Örebro, Sweden; Scholar in Residence, Nicholas School of the Environment, Duke University, Durham, NC, USA; Teresa Heinz Professor of Green Chemistry, and Director, Institute for Green Science, Department of Chemistry, Carnegie Mellon University, 4400 Fifth Avenue, Pittsburgh, PA 15213, USA; HEEDS, Environmental Health News, Charlotte, VA, USA; School of Molecular Biosciences, Center for Reproductive Biology, Washington State University, Pullman, WA 99164, USA; Graduate School of Nanobioscience, Yokohama City University, Yokohama, Kanagawa, 236-0027, Japan; Centre for Pollution Research and Policy, Brunel University London, Uxbridge UB8 3PH, UK; Environmental Health Sciences, Charlottesville, VA, USA; Department of Chemistry, Carnegie, Mellon University, Pittsburgh, PA, USA; Division of Biological Sciences, The University of Missouri, Columbia, MO 65211, USA; Centre Cavaillès, Ecole Normale Supérieure, Paris, France; Institut for Advanced Studies, Nantes, France; Department of Immunology, Tufts University School of Medicine, Boston, USA; Centre Cavaillès, Ecole Normale Supérieure, Paris, France; Department of Immunology, Tufts University School of Medicine, Boston, USA

**Keywords:** bisphenol A, BPA, European Food Safety Authority, European Medicines Agency, endocrine disrupting chemicals, EDCs, risk assessment

## Abstract

The European Food Safety Authority (EFSA) has revised their estimate of the toxicity of bisphenol A (BPA) and, as a result, have recommended reducing the tolerable daily intake (TDI) by 20 000-fold. This would essentially ban the use of BPA in food packaging such as can liners, plastic food containers, and in consumer products. To come to this conclusion, EFSA used a systematic approach according to a pre-established protocol and included all guideline and nonguideline studies in their analysis. They found that Th-17 immune cells increased with very low exposure to BPA and used this endpoint to revise the TDI to be human health protective. A number of regulatory agencies including the European Medicines Agency (EMA) have written formal disagreements with several elements of EFSA's proposal. The European Commission will now decide whether to accept EFSA's recommendation over the objections of EMA. If the Commission accepts EFSA's recommendation, it will be a landmark action using knowledge acquired through independent scientific studies focused on biomarkers of chronic disease to protect human health. The goal of this Perspective is to clearly articulate the monumental nature of this debate and decision and to explain what is at stake. Our perspective is that the weight of evidence clearly supports EFSA's proposal to reduce the TDI by 20 000-fold.

Bisphenol A (or BPA) is a chemical used in consumer products that has been the focus of international attention periodically over many years. But the scientific study of BPA has steadily continued to provide new insights into the toxicity of this chemical despite its on again–off again nature of news coverage. BPA is in the news again now because the European Food Safety Authority (EFSA) has formally proposed reducing the “safe level” of BPA in consumer products by 20 000-fold [1]. This change would essentially ban BPA from food contact materials and from many plastics used in a variety of consumer goods in Europe.

EFSA derived this new estimate of BPA toxicity by departing from their previous analyses—now they include in their analysis scientific findings derived from academic laboratories that focus on a precursor to disease—a specific immune cell type induced by BPA exposure. Trusting academic studies for risk assessment by regulatory agencies has been a highly contentious issue and not all regulatory agencies accept independent academic studies (see below). This means that safe levels of chemicals used in consumer goods are often set by studies mainly provided by the industries manufacturing the chemical. But EFSA's new risk assessment has based their analysis of BPA toxicity and risk to the exposed population on academic studies, as well as those traditionally used, and this has spawned a fight between EFSA and other regulatory agencies.

## “Guideline” vs “Nonguideline” Studies

We refer here to guideline studies as those in which the experimental design, endpoint measurement, and analysis have been developed and approved by national and/or international organizations. For instance, the Organization for Economic Co-operation and Development (OECD) publishes validated test guidelines describing the purpose of the study and essential elements of the experimental design. For illustration, the OECD test guideline for developmental neurotoxicity (OECD 426) focuses on body and brain weight, histological measurements of the brain, and other developmental landmarks. We refer to “nonguideline” studies as those using different designs and additional endpoints from those defined in regulatory guidelines. For instance, to test for neurotoxicity, an experiment may be designed to measure the ability of a chemical to alter hormone-dependent developmental events such as the number of oligodendrocytes in areas of white matter or the proper migration of populations of neurons. These nonguideline studies are designed to take advantage of fundamental knowledge about brain development and the timing of these events. The universe of nonguideline endpoints is defined by the body of basic scientific study of animal and human development and physiology. Often, but not always, regulators will prefer to use the results of guideline studies that have been performed in compliance with “Good Laboratory Practice” (GLP). GLP is a record-keeping structure that captures all the known elements of the conduct of the study but does not assess the scientific rigor of the design nor does it capture potential conflicts of interest [2].

## BPA Is a Prototypical Endocrine-Disrupting Chemical

BPA has long been a poster child for regulatory agencies’ difficulty with safety evaluations of chemicals that interfere with hormone systems—so-called endocrine-disrupting chemicals. The Endocrine Society—headquartered in Washington, DC, but with a global membership—has been intensely involved in work designed to ensure that regulations reflect modern endocrinological science. They do this by providing scientific insights into what hormones are, what they do, and how they do it [3]. BPA is best known as an estrogenic chemical, mimicking the action of the female hormone estrogen. But BPA also interacts with other hormone systems, including the hormone testosterone and the thyroid hormone system among others. It also acts as an inflammatory agent, affecting immune responses directly as well as indirectly via endocrine modes of action.

## Everyone Is Exposed Chronically to BPA

Even though BPA does not appear to stay in our bodies for long, nearly everyone has measurable amounts of BPA all the time because we are constantly being exposed to BPA, primarily through food and water as well as thermal paper receipts [4].

## What New Information Did EFSA Use?

Until now, EFSA has primarily used guideline GLP studies to evaluate the safety of BPA and establish a “daily tolerable intake.” In a break with this strategy, EFSA reviewed a wide range of studies and found that BPA exposure was related to a broad scope of harm, with the most sensitive outcome being an increase in the number of a specific type of immune cell that is known to be involved in inflammatory diseases and obesity (Th-17 cells). So EFSA revised their estimate of human risk from exposure to BPA by using the results of studies conducted by independent scientists in addition to the standardized studies normally conducted by industry.

Considering this, EFSA's proposal has drawn criticism from industry groups and the European Medicines Agency (EMA). The criticism by the EMA is important because it is an agency whose stated mission is to foster scientific excellence in the evaluation and supervision of medicines for the benefit of public and animal health in the European Union. EMA input is a necessary step in the acceptance of regulatory recommendations made by EFSA.

## EFSA's Opinion Targets the Conflict Between Regulatory Toxicology and Endocrinology

The divergent opinions about EFSA's draft proposal on BPA safety reflect the debate that has been ongoing for at least 3 decades [5]. The 3 main issues under debate are the definition of an “adverse effect,” the use of an “intermediate endpoint,” and what scientific studies should be selected for use in safety evaluations?

## What Is an Adverse Effect?

To protect public health, regulatory agencies focus on what adverse effects a chemical may have. However, this is more subjective than it sounds. For instance, the US FDA has only recently defined what they view as an adverse effect: “For an observed effect to be toxicologically relevant (ie, potentially adverse), a clear dose–response should be seen (eg, increasing the dose of a test substance causes an increase in the observed effect in the test subjects), and the observed effect should occur in both sexes of test species” [6].

But there are 3 major problems with this statement:

1. It does not tell us what kind of “endpoints” (eg, uterine weight, blood glucose, body weight gain, brain development) should be considered adverse for use in safety determinations.
2. The 400-year-old “dose makes the poison” concept requiring an ascending relationship between “dose” and “response” is not supported by our understanding of endocrine systems. Hormones all exhibit a phenomenon called “high-dose inhibition,” which is why a hormone (eg, leuprolide acetate, a gonadotropin-releasing hormone agonist) that is used at low doses to stimulate reproduction in women can be used at a high dose to block testosterone production in men with prostate cancer. Therefore, we cannot ignore low-dose effects if there are no observed effects—or if there are different effects—at high doses.
3. The concept that the effect must be observed in both sexes is ignorant of biology; men and women circulate different levels of hormones, and a signature feature of endocrine-disrupting chemicals is that adverse effects in males and females are rarely the same. As an obvious example, cancers of the uterus, ovary, and testis (for instance) will always be sex specific.

In this case, EFSA used the effect of BPA on the number of Th-17 cells as the most sensitive adverse effect. The EMA and other agencies disagreed with this opinion arguing that the number of Th-17 cells do not represent an apical endpoint. The word “apical” here refers to measuring something that is the consequence of a chain of events triggered by chemical exposure resulting in the final adverse outcome. EMA therefore makes the argument that the BPA-induced increase in Th-17 cells is not causally related to the “apical” endpoint of allergies.

## The Intermediate Endpoint on the Path to Adverse Outcome

The EMA argued that there is no scientific support for a causal relationship between this immune cell type and immune-dependent allergies; therefore, Th-17 cells cannot be used as a surrogate for the adverse effect of immune-dependent allergies. However, EFSA countered that an increase in Th-17 cells is causally related to the inflammatory pathogenesis leading to diseases like psoriasis and asthma; therefore, the BPA-induced increase in Th-17 cells represents an appropriate intermediate endpoint on the path to an adverse outcome. In addition, EMA argued that the specific immune effect observed by EFSA was not observed by the US-based CLARITY-BPA study. EFSA countered that this reasoning ignores the fact that CLARITY-BPA did not examine these immune effects.

## Uncertainty in the Face of Intermediate Endpoints

The EMA stated that, “EMA considers indeed there is insufficient evidence to conclude on the biological plausibility of a causal association between Th-17 cell percentage and immune disorders, especially given that a causal link has not been demonstrated in a study in animals or humans.” Notwithstanding EMA's confusion about the relationship between Th-17 cells and disease endpoints, EMA's questioning of the use of an intermediate endpoint in lieu of an “apical” endpoint is dangerous. Public health will not be served if we regulate chemical exposure based on (for instance) retinopathy instead of on serum glucose. We know enough about the relationship between these 2 events (glucose and retinopathy). Likewise, public health will not be served if we must regulate chemical exposures based on cognitive deficits in children instead of based on the ability of a chemical to cause a decrease in serum thyroid hormone. We know enough about the relationship between these 2 events (maternal thyroid hormone and offspring cognitive development). The cost of inaction—or delayed action while waiting for the appearance of end-stage disease—costs society billions of euros (or dollars) annually as well as impacting quality of life [7].

## What Scientific Studies Should Be Included for Safety Determinations?

The scientific enterprise today is more sophisticated than at any time in human history, but some regulatory agencies routinely exclude independent academic findings in preference to using those from guideline, GLP-compliant studies. However, GLP is not a guarantee for good (or competent) science [2]; GLP ensures that appropriate records are kept. GLP does not assure that the right questions are asked or that the experimental design is appropriate for the question. Most academic studies receive far more rigorous review than industry-based guideline studies because academic research typically has 2 quality filters: first, it must be sufficiently rigorous to qualify for government funding through a highly competitive peer review process at (for instance) the NIH in the US or INSERM in France. Second, once funded, academic research must pass peer-review in the publication process. Today's risk assessment process often selectively embraces industry-funded literature (eg, reports that industries present to regulatory agencies) that qualifies because of GLP compliance, even though these data are often unpublished and not available to the public.

Still, EMA states explicitly that they, “…require(s) studies are carried out in accordance with GLP…”. In contrast, EFSA used a systematic approach according to a pre-established protocol and included all guideline and nonguideline studies. Other regulatory agencies have no such requirement that data used in regulatory decisions be generated in studies compliant with GLP and it is reasonable and public health protective that EFSA embrace the body of scientific information.

## CLARITY-BPA Tells us About the Difference Between Guideline and Nonguideline Studies—and the Proponents of Each

The EMA refers in their arguments to data generated by the Consortium Linking Academic and Regulatory Insights on BPA Toxicity (CLARITY-BPA) that was developed to study the full range of potential health effects caused by BPA exposure comparing guideline compliant and academic studies. The “core study” was performed by an FDA laboratory, measuring guideline endpoints like organ weight and body weight and various organ histology. Then they sent various tissues (after concealing the identity of the treatments) to 14 different academic laboratories that measured endpoints related to prostate and mammary gland cancers, thyroid function and brain development, cardiovascular development, behavior and reproductive endocrinology, and others [8]. While the EMA argued that Th-17 cells were not affected by BPA in the CLARITY study, this measurement was not taken. Perhaps more telling is that EMA did not point out that many endpoints—including in both the core and the academic studies—were affected by the lowest dose of BPA including in brain, prostate, urinary tract, ovary, mammary gland and heart [8]. These findings fully support EFSA's current proposal of a 20 000-fold reduction in the tolerable daily intake.

## What Will the European Commission Decide?

EFSA's proposal now goes to the European Commission to decide whether to approve of the new BPA restriction or agree with EMA that the proposal is not scientifically justified. We strongly support EFSA's analysis and disagree with EMA's criticism on each count. Following the logic of EMA would require that we identify end-stage chronic disease as an “apical” endpoint. The use of an intermediate endpoint to base regulatory decisions is rational and health protective. Finally, to disregard independent scientific studies because of the arbitrary requirement for being GLP-compliant is a great disservice to the public, both in terms of public health and in terms of public investment. Thus, we strongly encourage the European Commission to accept EFSA's proposal without modification and to implement this proposal quickly.

## Disclosures

The authors declare that there are no conflicts of interest.

## Data Availability

No original data were generated for this Perspective.

## Abbreviations

BPA: bisphenol A
EFSA: European Food Safety Authority
EMA: European Medicines Agency
GLP: Good Laboratory Practice
OECD: Organization for Economic Co-operation and Development
TDI: tolerable daily intake
